# Neural Correlates of Effective Learning in Experienced Medical Decision-Makers

**DOI:** 10.1371/journal.pone.0027768

**Published:** 2011-11-23

**Authors:** Jonathan Downar, Meghana Bhatt, P. Read Montague

**Affiliations:** 1 Department of Psychiatry, University of Toronto and Toronto Western Hospital, Toronto, Ontario, Canada; 2 Beckman Research Institute, City of Hope Hospital, Duarte, California, United States of America; 3 Human Neuroimaging Laboratory, Virginia Tech Carilion Research Institute and Department of Physics, Virginia Tech, Roanoke, Virginia, United States of America; 4 The Wellcome Trust Centre for Neuroimaging, University College London, London, United Kingdom; Kyushu University, Japan

## Abstract

Accurate associative learning is often hindered by confirmation bias and success-chasing, which together can conspire to produce or solidify false beliefs in the decision-maker. We performed functional magnetic resonance imaging in 35 experienced physicians, while they learned to choose between two treatments in a series of virtual patient encounters. We estimated a learning model for each subject based on their observed behavior and this model divided clearly into high performers and low performers. The high performers showed small, but equal learning rates for both successes (positive outcomes) and failures (no response to the drug). In contrast, low performers showed very large and asymmetric learning rates, learning significantly more from successes than failures; a tendency that led to sub-optimal treatment choices. Consistently with these behavioral findings, high performers showed larger, more sustained BOLD responses to failed vs. successful outcomes in the dorsolateral prefrontal cortex and inferior parietal lobule while low performers displayed the opposite response profile. Furthermore, participants' learning asymmetry correlated with anticipatory activation in the nucleus accumbens at trial onset, well before outcome presentation. Subjects with anticipatory activation in the nucleus accumbens showed more success-chasing during learning. These results suggest that high performers' brains achieve better outcomes by attending to informative failures during training, rather than chasing the reward value of successes. The differential brain activations between high and low performers could potentially be developed into biomarkers to identify efficient learners on novel decision tasks, in medical or other contexts.

## Introduction

Learning effectively from experience is a daunting task for any organism. For every good or bad outcome, there are an immense number of potential causes and associations to be considered. For many decisions, it can be nearly impossible to pick out the few relevant factors from the many irrelevant factors, even with extensive experience. A major stumbling block for learning in these multi-dimensional environments is the tendency to form spurious beliefs: i.e., to attribute a causal role to factors that have no actual bearing on the outcome.

The formation of spurious beliefs is universal, from Skinner's observations of superstitious pigeons [1] to an athlete's belief in a lucky hat. In some situations, these beliefs are essentially harmless; by-products of learning mechanisms, but in other settings their impact can be severe. For example, spurious associations can have literal life-or-death consequences when they affect the complex decisions made by physicians. These expert decision-makers must extract and distill relevant features from a myriad of tests, symptoms, and personal histories, and employ these features to make critical medical decisions. Consequently, it is important to understand how spurious associations form and how they can bias subsequent decisions.

Previous studies have examined the neural basis of associative learning (and in particular, prediction-error models of learning) in non-physicians performing pseudo-medical decision-making tasks. These studies have identified the dorsolateral prefrontal cortex (DLPFC) as a key region whose activity correlates with the learning of causal relationships by coding for the unsigned prediction error at an outcome, and adjusting existing beliefs based on this new information [2], [3]. These findings suggest the hypothesis that when this region fails to distinguish correctly between important and unimportant associations, spurious learning and false belief formation ensue [4]. In the extreme case of psychosis, disordered functioning of the DLPFC and its striatal counterpart regions may underlie the inability to reverse previously held beliefs in the face of contradictory information, thus contributing to the delusions of schizophrenia and other psychotic disorders [5], [6]. A similar process could also operate during spurious belief formation among medical experts. However, so far, the neural correlates of medical decision-making in physicians have yet to be explored.

Aside from the DLPFC, other brain regions also appear to have important roles in learning and decision-making. Where the DLPFC codes unsigned prediction error, the striatum appears to code for the signed prediction error between expected and actual reward outcomes [7], [8], [9], [10], [11], [12], [13]. The nucleus accumbens (NAC) in particular appears to code specifically for reward and often shows anticipatory activations in expectation of rewards [14]. This anticipatory activation has been linked to the placebo effect, where this anticipation is able to produce subsequent physiological effects [15]. The association between NAC activity and both the anticipation and experience of reward suggests that the increased activity in advance of expected successes could contribute to confirmation bias and success-chasing. Finally, the inferior parietal cortex plays an important role in associative learning, by identifying salient events in the sensory environment, whether the salience is driven by top-down factors such as behavioral relevance, or bottom-up factors such as novelty [16], [17]. Hence, biases in individual physicians' learning behavior might also be reflected in the activity of these areas, in addition to the DLPFC.

Based on these previous findings, we aimed to test three hypotheses in the present study. First, among medical experts, individuals who develop spurious associations during learning should show disproportionately large adjustments of beliefs after rewarding or salient events, as observed in their decision-making behavior. Second, the individuals who develop spurious associations during learning should show a distinct pattern of activation in the DLPFC and inferior parietal lobe in response to outcomes, compared to those who do not. Third, the individuals who develop spurious associations should show greater activity in the NAC in anticipation of rewarding outcomes, compared to those who do not.

To test these hypotheses, we used functional magnetic resonance imaging (fMRI) to study neural activation in a population of 35 experienced physicians while they learned to decide between two fictional treatments in a series of virtual patient encounters. Next, we collected behavioral data on the physicians' choices between the two treatments in a second series of virtual encounters, to identify high- and low- performers based on their ability to select the optimal treatment for each encounter. We then used both behavioral and neuroimaging measures to characterize the differences between high- and low-performers during learning. To our knowledge, this is the first study to examine brain activation in physicians, during learning and decision-making within their domain of expertise.

## Materials and Methods

### Ethics Statement

All procedures were conducted with the approval of the Institutional Review Board of Baylor College of Medicine. Written consents were obtained from all subjects.

### Overview

Subjects were instructed that they would select treatments for a series of simulated patients with acute myocardial infarction (MI) in an emergency room setting. For each patient, they viewed a simplified, 6-factor clinical history before selecting one of two fictional treatments (‘*Levocyte*’ and ‘*Novotrin*’). They were instructed that both agents had some efficacy, but that they would need to learn by experience whether one medication was more effective than the other overall, or for certain types of patients. Unknown to subjects, both medications had equal success rates of 50% overall. However, one medication, Drug A, (*Levocyte* for 18 subjects, *Novotrin* for the rest) had a 75% success rate in patients with diabetes, but only a 25% success rate in patients without diabetes. For Drug B, the opposite was true. 5 other plausibly relevant factors were also presented for each case: age, gender, symptom duration, history of smoking, or history of previous MI. However, aside from diabetes status, none of the other factors was actually relevant to treatment efficacy. Diabetes status is plausibly powerful predictor of outcome in this study population, as physicians are aware that a history of diabetes confers roughly the same risk as a history of previous MI in predicting future MI and associated mortality [18].

While the learning problem was presented in this familiar frame to enable learning in the multi-dimensional space, the use of fictional treatments ensured that subjects had neutral prior beliefs about their efficacy. Patient history factors were chosen so that they conferred similar risk for MI, but did so through distinct mechanisms, in order to avoid excessive variation in subjects' prior assumptions on how patient history might affect treatment efficacy.

### Participants

The study included 35 physicians from a variety of non-surgical specialties (full demographic information is presented in Table S1). All participants were affiliated with Baylor College of Medicine. Participants with a history of active neurological or psychiatric illness, including substance dependence, head injury with loss of consciousness >10 min, or current use of psychotropic medications were excluded from the study.

### Decision-Making Task

**Figure 1:**
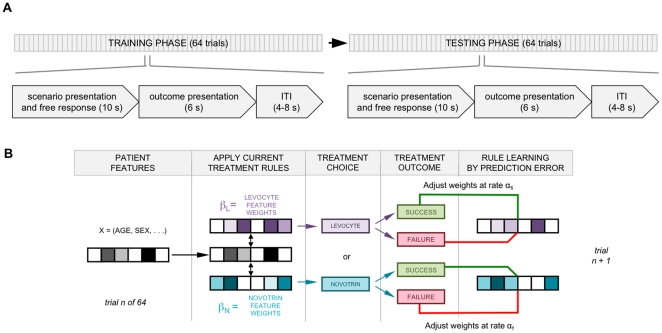
Task Design and Learning Model. **A:** The task consisted of a training phase followed by a testing phase. During the training phase, subjects proceeded through a fixed sequence of 64 patient encounters. They had 10 s to select one of two fictional medications. A red frame appeared around the selected medication at the time of the key press. At the end of the 10 s, they saw the outcome of their selection: either ‘SUCCESS: MI aborted’ or ‘FAILURE: No response’. The outcome remained on the screen for 6 s. A fixation cross then appeared during an intertrial interval of 4–8 s before the next encounter. The testing phase used a different permutation of encounters, and the presented outcome was always the neutral statement ‘Selection recorded’. **B:** In the modified Rescorla-Wagner learning model, subjects predict the efficacy of the two treatments using association rules, modeled as weighting vectors for each of the 6 patient features (plus a constant term). Following treatment choice and outcome presentation, the weighting vector for the selected treatment is adjusted according to Δβ_drug_ = α_outcome_·PE·X, where PE is the prediction error. One can therefore derive separate learning weight constants for successes and failures, using each individual's choices during testing, in combination with each individual's choices and associated outcomes during training.

Subjects first proceeded through 64 patient encounters in a Training Phase (Figure 1a). In each encounter, they saw six items of information about the patient: age, sex, hours from symptom onset, presence or absence of smoking history, previous MI, and diabetes. Subjects had 10 seconds to select a treatment. After selecting a treatment, they were presented with a binary outcome: ‘SUCCESS: MI aborted’ or ‘FAILURE: No response’ (for illustration, see Figure S1). The outcome screen appeared for 6 seconds, followed by a randomly determined 4–8 second inter-trial interval drawn from a uniform distribution. Next, they proceeded through a permutation of these 64 patients in a Testing Phase. To avoid further learning effects, in the Testing Phase, the outcome was the neutral phrase ‘Selection recorded’ (Figure 1a).

Both sequences used a counterbalanced design with respect to all patient features. The outcomes for each of the two choices on each trial were predetermined, although subjects were not informed of this.

### Neuroimaging

We acquired anatomical and functional images using 3.0T Siemens Trio MRI scanners. Padding and head restraints minimized head movement during image acquisition. Anatomical imaging used an MPRage sequence to obtain high-resolution, T1-weighted images of the whole head. Functional imaging used an EPI sequence with a repetition time (TR) of 2000 ms, echo time (TE) of 30 ms, 90° flip angle, 220 mm field of view, 64×64 pixel image matrix, and 34×4 mm slices for measurement of the blood oxygenation level-dependent (BOLD) effect [19], [20], [21]. Functional image slices were oriented 30° superior-caudal to the plane through the anterior and posterior commissures, to minimize signal dropout due to magnetic field inhomogeneities at air/tissue interfaces. The resulting functional image voxels had dimensions of 3.4×3.4×4.0 mm. Subjects viewed visual stimuli on a rear-projection screen using an angled mirror attached to the head coil and made responses using a two-key, fiber-optic button box.

We performed functional data preprocessing and analysis using SPM8. Preprocessing included within-subject motion correction, coregistration of anatomical and functional images, spatial normalization to the standard MNI template brain, spatial smoothing using a Gaussian kernel of 6 mm full-width at half-maximum, and high-pass filtering in the temporal domain at 1/128 Hz. Preprocessed voxels were 4×4×4 mm.

### Analysis of Behavioral Data

We used a logistic regression model of drug choice in the Testing Phase, using the 6 patient history factors as predictors to obtain an objective measure of subject treatment algorithms. Predictors were normalized to mean = 0 and standard deviation = 1 before analysis, to enable effect size comparisons.

In addition, subjects completed an exit questionnaire in which they explicitly described their own treatment algorithms in written form. Authors JD and MB independently reviewed the questionnaires to assess subjects' self-reported treatment algorithms. Inter-rater agreement was strong (*r* = 0.90); discrepancies were resolved by consensus.

### Analysis of Functional Data

For the Training Phase, responses to each task component were identified using general linear models with four conditions: scenario presentation, decision period, treatment selection keypress, and outcome presentation. These models included conditions for the interaction of decision period×time, and the parametric modulation of outcome presentation. In the first model we parameterized the outcome regressor with the simple binary success/failure variable, in the second we parameterized outcome by the implied signed prediction error and in the final model with parameterized the outcome with the unsigned prediction error. Regressors were all constructed by convolving stick functions at the relevant times with a canonical hemodynamic response function and its time and dispersion derivatives. Second level analyses were then performed in SPM8.

## Results

### Behavioral Performance

**Figure 2:**
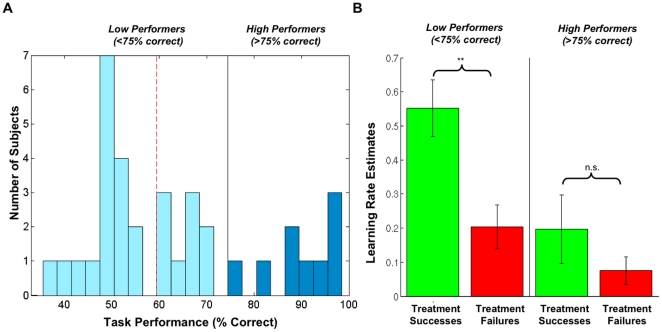
Behavioral Performance and Learning Model Estimation. **A:** Histogram illustrating bimodal distribution of task performance among subjects. A high-performing group exceeded 75% correct responses during testing, while a low-performing group fell below 75% correct responses. The threshold for above-chance performance (p<0.05) was >60% correct binary choices over 64 trials (dotted red line). **B:** Bar chart comparing the impact of treatment successes vs. failures on learning in low-performers and high-performers. Chart shows parameter means and standard errors for each group. Learning parameter estimates were calculated using a modified Rescorla-Wagner learning model. High performers made small but non-zero rule adjustments following both successes and failures, while low performers made large rule adjustments, and made significantly larger adjustments following successes than failures. In low performers, the median learning parameter estimate for failures was zero. Paired t-tests showed that low performers had significantly higher success learning rates than failure learning rates (p<0.001), while high performers showed no significant difference. In addition, a two-sample t-test showed that low performers had overall higher learning rates than low performers (p<.01).

The optimal treatment strategy would be to select Drug A for all patients with diabetes and Drug B for all other patients. However, the average performance was substantially worse than optimal (mean rate of optimal choices, 64% +/− 18% SD). 17 of 35 subjects chose the optimal drug at, or at worse than, chance levels (optimal choices ≤38 of 64; p>0.05, binomial distribution). Performance had a bimodal distribution, with the majority of subjects performing at, or slightly better than random and a minority performing significantly better than random. Using a k-means algorithm, we divided subjects into two clusters. 9 of 35 subjects fell into the high performing group, choosing the optimal drug in between 77% and 98% percent of Testing trials (Figure 2a; Table S2). The other 26 fell into the low performing group, choosing the optimal drug in between 38% and 70% of the trials. Using 3 instead of 2 clusters did not significantly change the characterization of the high performing group – which went from being the best 9 subjects to the best 8 subjects. It did, however, subdivide the low performing group into 2 groups: the 17 subjects who performed at or worse than random and 10 subjects who were better than random but not part of the high performing cluster.

Despite the poor performance of many of the subjects, the performance distribution is largely above chance levels, with half the subjects performing significantly better than random and only 1 subject performing significantly worse than random. This indicates that the group as a whole exhibited some learning on the task, and high performance was not an artifact of simply stumbling upon the correct rule. Notably, there was a significant negative correlation between subjects' number of optimal choices and years of clinical experience (r = −0.4, p<0.05; Tables S2 and S5).

### Spurious Rule Learning

Every subject reported using at least one of the 5 irrelevant factors in their treatment algorithm (mean irrelevant factors, 3.3 +/− SD 1.3). As an illustrative example, subject 30 described an algorithm of using Levocyte for females >55 or males <55 years old, but Novotrin for males >55 or females <55 years old. Subject 22 described using Levocyte in females, but Novotrin in patients presenting late after symptom onset, or in patients <60 *or* >75 years old, and “?” in smokers. Neither made any reference to diabetes status. Not one of the 35 subjects reported that the treatments appeared to have equal efficacy regardless of patient presentation. Not one of the 35 subjects reported guessing or choosing randomly during the Testing Phase.

We compared these subjective reports to the subjects' actual choices in the Testing Phase using a logistic regression model (Table S2). On this objective measure, diabetes status significantly predicted choice (*p*<0.05) in 14 of 35 subjects (including 7 of the 9 high performers), with 1 subject showing a significant predictive effect in the wrong direction. At the same time, one or more irrelevant factors significantly predicted choice (*p*<0.05) in 25 of 35 subjects. Diabetes status was the *largest* factor influencing choice in 11 of the 35 subjects (including all high-performers), all in the correct direction. This number is significantly higher than would be predicted if the dominant rule were chosen at random in each subject (p<0.01). No other factor dominated choice in a consistent direction in a significantly sized subset of subjects. Presence of a previous MI did dominate choice in 9 subjects, which was significant at p<0.05. However, the direction was inconsistent, with an association to Drug A in 5 subject and to Drug B in 4 subjects.

We also computed the optimal treatment strategy given each individual's set of outcomes during training, to test whether spurious rules were formed as a rational consequence of the particular outcomes each subject happened to see as a result of their choices during training. Only in subjects 1 and 3 did a single treatment-irrelevant factor inadvertently achieve predictive significance during training. Subject 1 still did not develop a significant treatment rule around this factor. Subject 3 did develop a rule around the factor, as well as 3 other confirmed irrelevant factors. Thus, the rules they formed from their experiences were indeed spurious, rather than *bona fide* reflections of the training outcome series (Tables S3 and S4).

### Learning Model Estimates

We used a modified Rescorla-Wagner [22] learning model to quantify subjects' learning rates separately for successes and failures. In this model, each drug's efficacy was approximated by a linear function of the patient features (bounded by 0 and 1 from above and below): 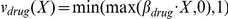, where *X* is the normalized vector of patient features plus a constant term. In each training trial where the subject chose a particular drug, its coefficients 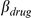 are adjusted according to 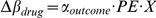. Here *PE* is the prediction error from that trial and 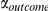 is the learning rate for the outcome type (either success or failure).

Each subject's final treatment rule is expressed as the 7-dimensional vector encompassing the relative influence of each factor on treatment choice, with values drawn from the logistic regression of choices on features during the Testing phase. Specifically, the logistic regression on drug choice during testing gives us a linearly scaled estimate of the difference between the two drugs values: *λ*⋅(*v_drug A_*(*X*)−*v_drug B_*(*X*)). We used this measurement to estimate the implied impact of successes and failures on subjects' final decision rules by finding the values for 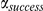 and 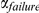 that minimized the angle between (*v_drug A_*(*X*)−*v_drug B_*(*X*)) and the objectively determined treatment algorithm (Figure 1b, Table S5). This method of estimation allowed us to avoid making the assumptions about how the subjects may have explored the state space during the training phase required by a standard maximum likelihood estimation. This was particularly important for this population since some subjects explicitly tried to sample the state space evenly (one subject actually chose drug A for the first half of the training phase and drug B for the second half), so their decisions during the training phase were not necessarily informative of their valuations. Results of this estimation along with goodness of fit measures are reported in table S5.

High performers showed relatively small learning rates, but learned relatively equally from successes and failures (t = 1.16, *p* = 0.28 in a paired t-test); all but 1 had strictly positive (i.e., non-zero) learning rates on both failures and successes. In contrast, low performers showed very large and asymmetric learning rates, learning significantly more from successes than failures (t = 4.12, *p* = 0.0004 in a paired t-test); 11 out of 26 low performers showed a learning coefficient of zero for failures (Figure 2b; Table S5). The size of the learning rates for low-performers was significantly higher than those for high performers (t = 2.92, p = .007 in a 2 sample t-test assuming unequal variances). Hence, our model identified a distinct learning profile for high performers as compared to low performers. Small but relatively symmetrical rule adjustments on each learning trial led to high performance, while large and asymmetrical adjustments led to poor performance.

As an additional confirmation of the validity of the model, we sought to determine whether we could predict the idiosyncratic, spurious algorithms formed by each low-performing subject using the computed learning rates for successes and failures, as applied to the subject's series of training encounters and outcomes. Using this approach, we were indeed able to predict each subject's final set of spurious treatment rules with good accuracy (Table S5). The error between the model-predicted and actual treatment algorithms is expressed as the angle between the vectors for the model-predicted and actual treatment algorithms. The mean error angle was 36.7 degrees in the 7-dimensional space. The probability of two random 7-dimensional vectors aligning as or more closely than 36.7 degrees is less than 0.05 (Table S5). In addition, we compared the fit of our modified model with the standard Rescorla-Wagner model, where learning rates are equal on successes and failures, on behavior during the Testing phase using the Akaike Information Criterion (AIC). Our model showed significantly improved fit as evidenced by lower AIC's in a paired t-test (Table S5, t = 4.47, p<0.0001).

### Neuroimaging Results

We hypothesized that activity in striatum and DLPFC should correlate with the learning asymmetries and performance differences described by our behavioral analysis, since both regions have been previously identified as coding for the signed and unsigned prediction error during formal associative learning. We also hypothesized that areas implicated in salience judgments, such as the inferior parietal cortex, should reflect subjects' learning biases.

**Figure 3:**
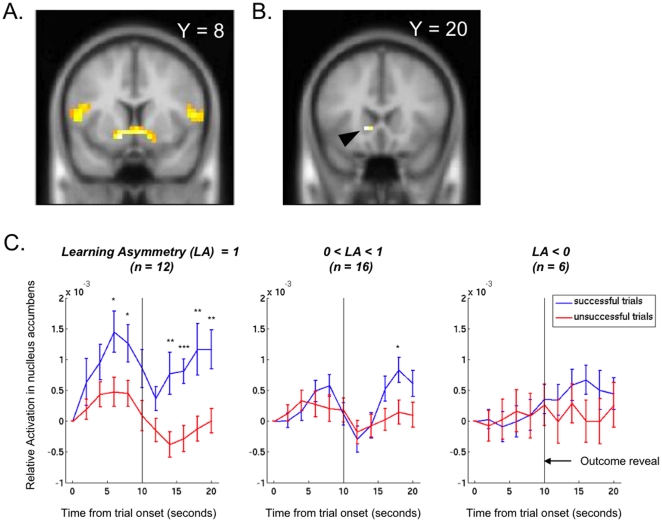
Ventral striatum correlates with prediction error at outcome in all subjects. Nucleus Accumbens correlates with learning asymmetry at trial onset. **A:** Activation from the analysis of correlates to prediction error across all subjects shown at p<0.001 uncorrected, Y = +8. **B:** Left nucleus accumbens correlates with learning asymmetry at trial onset, activation shown at p<0.001 uncorrected, Y = +20 (peak activation at (−14, 20, −2)). **C:** Timeseries of activation in left ventral striatum/nucleus accumbens separated by learning asymmetry and trial outcome: Success-chasers who completely ignore failure (left) show large anticipatory activation in the nucleus accumbens before both failures and successes, with even greater anticipation in advance of successes. They show even more significant differential activation to success vs. failure after the outcome reveal. People who over-weighted success but had positive learning rates on failures (middle) show some anticipatory activation in the nucleus accumbens before outcome reveal, but the area does not seem to significantly differentiate between successes and failures until after outcome is revealed. Finally, people who weighted failures more than success (right) show no anticipatory activation or significant differentiation between successes and failures after outcome is revealed. Significant differences in the timeseries are marked for p<0.05 (*), p<0.01 (**), and p<0.001 (***) in a 2 sample t-test. Note that timeseries are time-locked to scenario presentation time.

**Figure 4:**
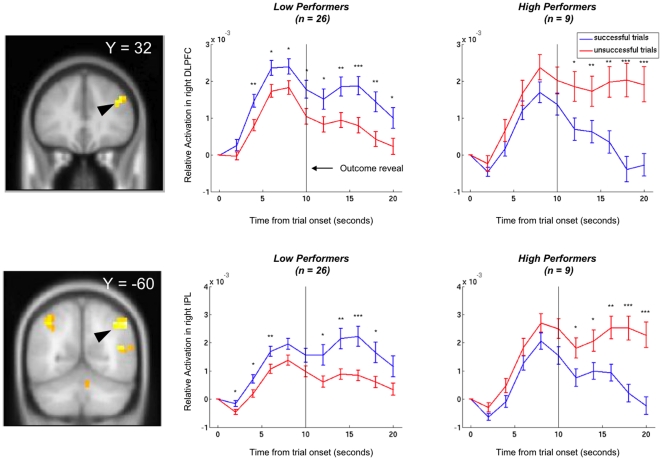
Significant interactions between response to success vs. failure and performance. **A:** Coronal slice shows dorsolateral prefrontal region with different responses to treatment success vs. failure in high vs. low performing subjects (left). Region-of-interest plots show timeseries of relative activation in the area on successful (blue) and unsuccessful (red) trials for high and low performing subjects (right). **B:** Coronal slice shows differential responses to success and failure in the in the inferior parietal lobule (IPL). Region-of-interest plots show timeseries of activation in the right IPL on successful (blue) and unsuccessful (red) trials for high- and low-performing subjects. Timeseries error bars indicate standard error. Significant differences at each timepoint are marked for p<0.05 (*), p<0.01 (**), and p<0.001 (***) in a 2-sample t-test. Note that timeseries are time-locked to scenario presentation time.

As expected, bilateral ventral striatum activity was highly correlated with the signed prediction error (Figure 3a, p<0.01 corrected for false-discovery rate (FDR) across the whole brain, Table 1). However, contrary to expectations, the DLPFC did not show a significant correlation with unsigned prediction error in this study across all subjects. However, the activity of this area showed considerable heterogeneity across subjects. Specifically, the right DLPFC showed significant effects of the interaction of group (low vs. high performers) with reaction to success vs. failure (p<.05, whole-brain FDR-corrected). While high performers showed significantly greater activation in the area after failures, low performers showed the reverse pattern of slightly greater activation in the area after successes (Figure 4a, Table 2).

**Table 1:** Brain regions showing correlation with signed prediction error during the outcome phase of the learning trials across all subjects.

Brain	Brodmann	Number of	MNI Coordinates			Peak
Region	Area	Voxels	X	Y	Z	Z-Score
Bilateral Ventral Striatum	–	27	−14	8	−10	5.27
L Middle Temporal Gyrus	37	36	−58	−52	−2	4.71
R Cerebellum	–	19	22	−64	−26	4.63
R Superior Parietal Lobule	7	11	18	−56	58	4.39
Right Lateral Prefrontal Cortex	44	9	54	8	10	4.38
L Angular Gyrus	39	46	−46	−68	22	4.27
Left Lateral Prefrontal Cortex	44	11	−50	4	14	4.23

Identified regions are based on voxelwise p<0.01 (corrected for the false discovery rate) with a minimum cluster size of k = 5 (320 mm^2^).

(p<0.0001 uncorrected, p<0.01 corrected for FDR, k>5).

**Table 2:** Brain regions showing differential activity for treatment success vs. failure among high (>75% optimal choices) and low (<75% optimal choices) performing subjects.

Brain	Brodmann	Number of	MNI Coordinates			Peak
Region	Area	Voxels	X	Y	Z	Z-Score
R Temporoparietal Junction	39	26	42	−64	18	4.52
R Inferior Parietal Lobule	40	49	50	−60	46	4.44
Cerebellum	–	51	10	−72	−26	4.08
L Inferior Parietal Lobule	40	29	−42	−56	46	3.96
R Dorsolateral Prefrontal Cortex	9	11	42	32	30	3.80
R Middle Frontal Gyrus	6	5	34	8	54	3.72

Identified regions are based on voxelwise p<0.001 (uncorrected) with a minimum cluster size of k = 5. All regions shown survive correction for FDR at p<0.05 at peak voxel.

Coordinates indicate the location of each region's statistical peak, with respect to the anterior commissure, in millimeters, in the standard space of the Montreal Neurological Institute MNI152 anatomical template.

(p<0.001 uncorrected).

The interaction of group by response to success vs. failure was also significant in the inferior parietal lobule bilaterally (p<0.05 corrected for FDR over the whole brain). Time courses in the inferior parietal lobule followed the same pattern as the right DLPFC, with low performers showing increased activation after successes and high performers showing increased activations after failures (Figure 4b). The complete set of regions showing a significant interaction of group by outcome type is given in Table 1b.

As hypothesized, activity in NAC correlated with ‘success-chasing’ in a between-subjects analysis. To identify success-chasers, we defined each subject's learning asymmetry as 
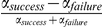
. Subjects with positive learning asymmetries over-weighted positive outcomes relative to failures during their learning process, consistent with success-chasing. Subjects with learning asymmetries of 1 ignored failures entirely. Subjects with negative learning asymmetries, conversely, adjusted their beliefs more after negative outcomes, consistent with failure-avoidance.

We found that increased activity in the left NAC at trial *onset*, before the outcome revealed, correlated significantly with learning asymmetry in this task (Figure 3b, peak voxel at (−14, 20, −2), z = 3.69, p<0.05 corrected for multiple comparisons in the small volume around the ventral striatum – a sphere of radius of 20 mm around (0, 8, −10)). Examination of the time-courses of activation from this area yielded two notable findings (Figure 3c). First, subjects showing the most success-chasing (with learning asymmetries of 1) showed significant anticipatory activation in the area for all trials, well before the outcome of the trial was revealed. Furthermore, this anticipatory activation was significantly larger prior to successful outcomes. Secondly, compared to the other subjects, the success-chasing subjects showed a significantly larger activation differential for successes versus failures after the outcome was revealed. In contrast, subjects with lower positive learning asymmetries showed less anticipatory activation, with no significant differential before the outcome was revealed and a much smaller differential between successes and failures after the outcome was revealed. Subjects with negative learning asymmetry showed no significant differential activation in the NAC during either phase of the encounter.

## Discussion

In this study we identified behavioral and neural characteristics of physicians who were adept at learning from experience. High performers learned from both successes and failures, and made smaller rule adjustments after feedback. Conversely, low performers learned disproportionately from successes, and made larger rule adjustments. Nearly half of the physicians performed at chance levels or worse, even after 64 training encounters. This result is particularly striking given that the difference in efficacy between the two treatments was intentionally made very large, at 75% versus 25% when diabetes status was taken into account. For comparison, the difference in real-world mortality between placebo and combined aspirin/streptokinase treatment for acute MI is only 13.2% versus 7.2% [23]. The suggestion from these results is that for most physicians, clinical experience alone may be inadequate for forming reliable heuristics about most real-world treatments, even when the differences in efficacy are large.

Spurious learning was also widespread in this study. On behavioral measures, more than two-thirds of physicians robustly incorporated spurious associations into their treatment algorithms. Overall, subjects were nearly twice as likely to invent a spurious rule as to detect the correct one. The high rate of spurious rule formation, in conjunction with the tendency to ignore failures, suggests how different experts might come to disagree vehemently about which factors are most relevant to decision-making, based on their personal experiences. The implication is that experiential learning alone is likely to capture irrelevant as well as relevant factors for guiding decisions, even among experts working within their domain of expertise.

Notably, years-of-experience was as strong a predictor of poor performance as were the number of spurious rules (r = 0.4 for each). Years-of-experience was also moderately (but not significantly) correlated with aggregate speed of learning (r = .24). The poorer performance of more experienced physicians on this task could conceivably reflect differences in training, temperament, or cognitive style. Although the present study was not designed to explore these possibilities in detail, understanding the basis of the experience-dependent decline in performance would be an important area for future investigation.

On neuroimaging, high- and low-performing subjects showed different patterns of activity in the inferior parietal cortex, and right DLPFC: brain areas with well-established roles in salience representation, associative learning, and the coding of prediction error [4], [16]. Low performers, who included irrelevant factors in their algorithms, showed stronger prefrontal and parietal activation for successes than for failures. Conversely, high performers showed stronger prefrontal and parietal activation after treatment failures than successes. The profile of activation in inferior parietal areas related to attention and salience [24] similarly suggest that while low-performers pay special attention to successes, high performers attend more to failures during learning.

The right DLPFC has a well-established role in learning causal relationships [2], [3]. The present study extends these previous findings to medical learning and decision-making in expert physicians, participating in a medically-framed decision-making task. While the present study did not detect significant correlations between unsigned prediction error and DLPFC our findings are consistent with the underlying hypothesis that the right DLPFC drives rule readjustment [2], [5]. Specifically, the differential activation of the area after successes in low performers, and after failures in high performers, does reflect the learning biases that characterize the two groups (Figure 4). The present study suggests that engaging these mechanisms following only successful predictions leads to inaccurate rule formation, and poorer predictions in the future, as spurious rules progressively accumulate.

Notably, the profile of DLPFC activation in low performers on this task bears a striking similarity to that seen during false-belief formation in pathological settings, such as in individuals with psychotic illness [6] or ketamine-induced psychosis in healthy subjects [5]. Thus, the existing literature on the role of the DLPFC in associative learning may have important implications not only for psychiatric patients, but also for the medical decision-makers who treat them.

If learning preferentially from successes is such an ineffective strategy, then why is it so pervasive even among experts operating in their domain of expertise? Here, the reward value of successes offers one possible explanation. Success-chasing, as measured by learning asymmetry, correlated significantly with anticipatory activity in the left NAC at trial onset (Figure 3). Notably, both this anticipatory activation and activation subsequent to outcome revelation showed significant differences between successful and unsuccessful trials in complete success-chasers (those with learning asymmetries of 1). In contrast, those who learned more from failures (with learning asymmetries less than 0) showed no anticipatory activation at all (Figure 3) and no significant differences in activation between successes and failures either before or after outcome was revealed.

These results support an interpretation of confirmation-bias among success-chasers: the confirmation of an expected reward leads to increased signal in the NAC in these subjects. This profile of activation is particularly interesting in light of evidence showing that activation in the NAC correlates with susceptibility to the placebo-effect [15], another example of confirmation bias in a completely different context. Whereas in the placebo effect the expectation of and effective treatment can lead to the alleviation of symptoms, here the expectation of reward appears to be amplifying each decision-makers response to reward both physiologically in the NAC itself, and behaviorally in there adjustments of their underlying beliefs.

Why would success-chasing be so prevalent despite its drawbacks? The ventral striatum and nucleus accumbens is known to play a key role in motivation and the prediction of a wide variety of rewards: juice, consumer goods, monetary gains, and gains in social reputation [8], [14], [25], [26]. The anticipatory activation of this region among asymmetric learners in the present study suggests that success-chasers may be excessively motivated by the reward value of a successful outcome. In their efforts to maximize successful outcomes during training, success-chasers may be paradoxically sabotaging their ability to learn effectively from past experience.

Taken together, the behavioral and neuroimaging results suggest that success-chasing and confirmation bias may underlie the relative pervasiveness of premature, asymmetric learning and the resultant poor performance of the majority of physician subjects in the present study. The general human bias towards confirmation over disconfirmation in hypothesis-testing has been extensively documented in a variety of non-medical contexts, such as the Wason Card Task [27]. Conversely, the necessity for disconfirmation learning in empirical investigations is a key principle identified by the philosopher of science, Karl Popper [28]. Conceivably, providing medical professionals with formal training in disconfirmation learning could improve their ability to learn effectively from clinical experience in real-world settings. Exploring this possibility would be an important area for future research.

In conclusion, the results of this study show distinct patterns of learning, both behaviorally and neurally, between effective and ineffective learners among physicians making decisions in a medically framed learning task. The tendency to chase successes and ignore failures provides a simple computational model of how spurious beliefs might be formed, and how different individuals seeing similar data might learn very different sets of associations. The neural differences observed could conceivably be developed into useful biomarkers for essential differences in individual learning styles. These may in turn prove useful in identifying those individuals who can resist the impulse to chase successes, and hence learn most effectively from experience. Finally, we note that although this study focused upon the specific case of medical decision-making, the findings may be also be relevant to many other fields in which experts must make high-stakes decisions by drawing upon personal experience.

## Supporting Information

Figure S1 **Illustration of scenario and outcome presentation.** At the beginning of each trial, the participant saw a simplified case history and were given up to 10 s to make a choice between the two possible treatments, which were presented with fictional names and logos. When the participant made a selection, a red box appeared around chosen treatment. At the end of the 10 s period, the outcome appeared at the bottom of the screen and remained for 6 s, followed by a variable fixation period and then the next trial in the series.
(DOC)

Table S1 **Full Demographic Information.** Years of clinical experience are counted from the year MD was obtained, less any years' leave for research, family, or other reasons. M, male; F, female.
(DOC)

Table S2 **Behavioral Performance Measures.** For behavioral data, DM and spurious rules are considered present if the presence of a factor predicts the subject's choice at *p*<0.05 in a logistic regression model. Yes (R) denotes the presence of a DM rule in the incorrect (reversed) direction. DM, diabetes mellitus.
(DOC)

Table S3 **Significance of factors in the Logistic Regression Model of Factors (Rules) Influencing Individual Subjects' Treatment Choices.** Table presents coefficients and *p*-values (below in italics) indicating the significance of each predictive factor,,in a logistic regression model of treatment choice, in each subject (as described in *Methods*). Coefficients shown here describe the probability that the subject would choose Drug A. Subject 18 chose the same medication for all patients during the Testing Phase. DM, diabetes mellitus. High performers are highlighted in bold.
(DOC)

Table S4 **Assessment of significant predictive factors in the training encounter set for each individual subject.** Although the training scenario sequence and the outcome of each of the two available choices was held fixed across all subjects, each subject still made a unique sequence of choices and thus saw a unique set of outcomes. Theoretically, a subject could have encountered a statistical difference in efficacy with, for example, smoking status, simply through chance sampling of the fixed outcomes. Hence, we assessed whether subjects' treatment rules inadvertently reflected real trends in the Training Phase. For each subject, we performed another logistic regression of all 6 factors against Training Phase outcomes for each drug. This allowed us determine, for each subject's unique set of outcomes, whether the two treatment choices actually did have significantly different efficacy as predicted by diabetes or any of the other patient factors. This table presents *p*-values indicating, for each factor, the significance of the observed differences in efficacy between the two medications, based on the specific sequence of choices and outcomes seen by each individual subject during training. P-values<0.05 are indicated in boldface. This model confirmed that diabetes status predicted optimal choice (*p*<0.05) in 33 of 35 subjects' training sets. Subject 10 mostly chose a single drug for diabetic patients during training, so would have been unable to determine differential efficacy. Only in subjects 1 and 3 did a single treatment-irrelevant factor inadvertently achieve predictive significance during training. Subject 1 still did not develop a significant treatment rule around this factor. Subject 3 did develop a rule around the factor, as well as 3 other confirmed irrelevant factors.
(DOC)

Table S5 **Learning Rate Estimates For High- versus Low Performers.** Estimated learning rates for high-performing and low-performing subjects, using the modified Rescorla-Wagner (RW) model as described in *Methods*. Subjects are ordered by percentage of optimal selections during the Testing Phase. All high performers showed positive learning rates from treatment failures as well as successes. 50% of low performers showed zero learning rates from failures. Model errors reflect the difference between the treatment algorithm predicted by the RW model with the learning rates as shown, and the actual algorithm as measured by the logistic regression model of treatment choices during the Testing Phase. Errors are expressed as the angle between normalized 7-dimensional vectors corresponding to the two algorithms, in degrees. AIC's are reported for both the adapted Rescorla-Wagner Model with asymmetric learning and a traditional Rescorla-Wagner Model. Subject 18 selected the same treatment for all patients, so learning rates could not be estimated.
(DOC)
