# Supplementary material for: Neural Correlates of Effective Learning in Experienced Medical Decision-Makers
Source: PLoS One. 2011 Nov 23;6(11):e27768. doi: 10.1371/journal.pone.0027768 (PMC3223201; doi:10.1371/journal.pone.0027768)
Supplement: Figure S1 — Illustration of scenario and outcome presentation. At the beginning of each trial, the participant saw a simplified case history and were given up to 10 s to make a choice between the two possible treatments, which were presented with fictional names and logos. When the participant made a selection, a red box appeared around chosen treatment. At the end of the 10 s period, the outcome appeared at the bottom of the screen and remained for 6 s, followed by a variable fixation period and then the next trial in the series. (DOC) [file pone.0027768.s001.doc]

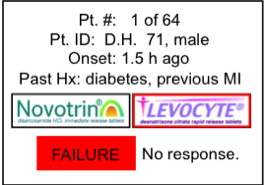


**Supplementary Figure S1: Illustration of scenario and outcome presentation.**

At the beginning of each trial, the participant saw a simplified case history and were given up to 10 s to make a choice between the two possible treatments, which were presented with fictional names and logos. When the participant made a selection, a red box appeared around chosen treatment. At the end of the 10 s period, the outcome appeared at the bottom of the screen and remained for 6 s, followed by a variable fixation period and then the next trial in the series.
