# Supplementary material for: Neural Correlates of Effective Learning in Experienced Medical Decision-Makers
Source: PLoS One. 2011 Nov 23;6(11):e27768. doi: 10.1371/journal.pone.0027768 (PMC3223201; doi:10.1371/journal.pone.0027768)
Supplement: Table S1 — Full Demographic Information. Years of clinical experience are counted from the year MD was obtained, less any years' leave for research, family, or other reasons. M, male; F, female. (DOC) [file pone.0027768.s002.doc]

**Supplementary Table S1. Full Demographic Information.**

| **Subject** |  | **Sex** |  | **Age** |  | **Specialty** |  | **Experience** |
| --- | --- | --- | --- | --- | --- | --- | --- | --- |
| **#** |  |  |  |  |  |  |  | **(years)** |
|  |  |  |  |  |  |  |  |  |
| 1 |  | F |  | 45 |  | Geriatrics |  | 18 |
| 2 |  | M |  | 35 |  | Internal Medicine and Pediatrics |  | 11 |
| 3 |  | M |  | 42 |  | Emergency Medicine |  | 15 |
| 4 |  | F |  | 46 |  | Hematology/Oncology |  | 16 |
| 5 |  | F |  | 44 |  | Hematology/Oncology |  | 19 |
| 6 |  | F |  | 40 |  | Anesthesia |  | 13 |
| 7 |  | M |  | 38 |  | Interventional Cardiology |  | 9 |
| 8 |  | F |  | 29 |  | Endocrinology |  | 6 |
| 9 |  | M |  | 40 |  | Hematology/Oncology |  | 12 |
| 10 |  | F |  | 37 |  | Pediatrics |  | 9 |
| 11 |  | F |  | 56 |  | Gastroenterology |  | 21 |
| 12 |  | M |  | 39 |  | Neurology |  | 14 |
| 13 |  | M |  | 59 |  | Gastroenterology |  | 33 |
| 14 |  | F |  | 40 |  | Internal Medicine and Gastroenterology |  | 16 |
| 15 |  | F |  | 48 |  | Internal Medicine and Endocrinology |  | 16 |
| 16 |  | F |  | 34 |  | Pediatrics |  | 8 |
| 17 |  | M |  | 47 |  | Internal Medicine and Critical Care |  | 20 |
| 18 |  | M |  | 56 |  | Internal Medicine |  | 26 |
| 19 |  | M |  | 59 |  | Internal Medicine and Pediatrics |  | 14 |
| 20 |  | M |  | 36 |  | Radiology |  | 7 |
| 21 |  | F |  | 32 |  | Internal Medicine |  | 7 |
| 22 |  | M |  | 34 |  | Radiology |  | 7 |
| 23 |  | M |  | 39 |  | Interventional Radiology |  | 11 |
| 24 |  | F |  | 40 |  | Geriatrics |  | 14 |
| 25 |  | F |  | 57 |  | Developmental Pediatrics |  | 15 |
| 26 |  | M |  | 40 |  | Medical Informatics |  | 14 |
| 27 |  | M |  | 31 |  | Cardiology |  | 6 |
| 28 |  | F |  | 44 |  | Sports Medicine |  | 19 |
| 29 |  | F |  | 29 |  | Hospitalist |  | 7 |
| 30 |  | F |  | 34 |  | Pediatric Anesthesia |  | 6 |
| 31 |  | F |  | 31 |  | Emergency Medicine |  | 5 |
| 32 |  | M |  | 41 |  | Hematology/Oncology |  | 15 |
| 33 |  | M |  | 39 |  | Hematology/Oncology |  | 7 |
| 34 |  | M |  | 38 |  | Emergency Medicine |  | 5 |
| 35 |  | M |  | 30 |  | Critical Care |  | 4 |

Years of clinical experience are counted from the year MD was obtained, less any years’ leave for research, family, or other reasons. M, male; F, female.
