# Supplementary material for: Neural Correlates of Effective Learning in Experienced Medical Decision-Makers
Source: PLoS One. 2011 Nov 23;6(11):e27768. doi: 10.1371/journal.pone.0027768 (PMC3223201; doi:10.1371/journal.pone.0027768)
Supplement: Table S2 — Behavioral Performance Measures. For behavioral data, DM and spurious rules are considered present if the presence of a factor predicts the subject's choice at p<0.05 in a logistic regression model. Yes (R) denotes the presence of a DM rule in the incorrect (reversed) direction. DM, diabetes mellitus. (DOC) [file pone.0027768.s003.doc]

**Supplementary Table S2. Behavioral Performance Measures**

| Subject  # | # Correct  (max. 64) | Self-Report | | Regression | |
| --- | --- | --- | --- | --- | --- |
|  |  | DM Rule | Spurious  Rules | DM Rule | Spurious  Rules |
| 1 | 49 | Yes | 3 | Yes | 1 |
| 2 | 57 | Yes | 3 | No | 0 |
| 3 | 32 | Yes | 5 | No | 3 |
| 4 | 52 | Yes | 4 | Yes | 2 |
| 5 | 39 | Yes | 3 | Yes | 0 |
| 6 | 43 | Yes | 2 | No | 0 |
| 7 | 26 | Yes (R) | 3 | No | 0 |
| 8 | 61 | Yes | 4 | No | 0 |
| 9 | 63 | Yes | 2 | Yes | 0 |
| 10 | 43 | Yes | 1 | Yes | 2 |
| 11 | 35 | Yes (R) | 5 | No | 3 |
| 12 | 24 | Yes (R) | 5 | Yes(R) (((R) (R)( (R) | 0 |
| 13 | 28 | No | 2 | No | 2 |
| 14 | 44 | Yes (R) | 3 | Yes | 2 |
| 15 | 31 | Yes | 4 | No | 1 |
| 16 | 33 | Yes | 5 | No | 4 |
| 17 | 32 | Yes | 3 | No | 1 |
| 18 | 32 | Yes | 1 | No | 1 |
| 19 | 45 | Yes | 5 | Yes | 3 |
| 20 | 40 | Yes | 3 | Yes | 3 |
| 21 | 33 | No | 2 | No | 2 |
| 22 | 58 | Yes | 4 | Yes | 1 |
| 23 | 34 | Yes (R) | 2 | No | 3 |
| 24 | 36 | Yes | 5 | No | 2 |
| 25 | 31 | No | 1 | No | 0 |
| 26 | 39 | Yes | 4 | No | 1 |
| 27 | 63 | Yes | 2 | Yes | 0 |
| 28 | 31 | No | 3 | No | 2 |
| 29 | 62 | Yes | 1 | Yes | 0 |
| 30 | 32 | No | 2 | No | 2 |
| 31 | 33 | Yes (R) | 3 | No | 2 |
| 32 | 29 | Yes (R) | 4 | No | 4 |
| 33 | 43 | Yes | 2 | No | 1 |
| 34 | 56 | Yes | 4 | Yes | 1 |
| 35 | 41 | Yes | 2 | Yes | 2 |

For behavioral data, DM and spurious rules are considered present if the presence of a factor predicts the subject’s choice at *p* < 0.05 in a logistic regression model. Yes (R) denotes the presence of a DM rule in the incorrect (reversed) direction. DM, diabetes mellitus.
