# Supplementary material for: Neural Correlates of Effective Learning in Experienced Medical Decision-Makers
Source: PLoS One. 2011 Nov 23;6(11):e27768. doi: 10.1371/journal.pone.0027768 (PMC3223201; doi:10.1371/journal.pone.0027768)
Supplement: Table S3 — Significance of factors in the Logistic Regression Model of Factors (Rules) Influencing Individual Subjects' Treatment Choices. Table presents coefficients and p-values (below in italics) indicating the significance of each predictive factor,,in a logistic regression model of treatment choice, in each subject (as described in Methods ). Coefficients shown here describe the probability that the subject would choose Drug A. Subject 18 chose the same medication for all patients during the Testing Phase. DM, diabetes mellitus. High performers are highlighted in bold. (DOC) [file pone.0027768.s004.doc]

**Supplementary Table S3**: Significance of factors in the Logistic Regression Model of Factors (Rules) Influencing Individual Subjects’ Treatment Choices

| Subject | Overall Rx Preference | Age | Sex | Time of Onset | Smoking | Prev MI | DM |
| --- | --- | --- | --- | --- | --- | --- | --- |
| **1** | **2.02** | **0.33** | **1.65** | **-0.30** | **0.15** | **0.14** | **2.56** |
|  | ***0.001*** | ***0.453*** | ***0.002*** | ***0.497*** | ***0.731*** | ***0.745*** | ***0.000*** |
| **2** | **-6.12** | **1.74** | **-0.21** | **1.38** | **-0.85** | **-5.65** | **12.48** |
|  | ***0.525*** | ***0.135*** | ***0.819*** | ***0.130*** | ***0.329*** | ***0.579*** | ***0.376*** |
| 3 | 1.64 | 1.97 | 1.05 | 2.66 | 0.15 | 1.26 | 0.03 |
|  | *0.005* | *0.001* | *0.052* | *0.001* | *0.744* | *0.015* | *0.948* |
| **4** | **-1.98** | **-2.31** | **0.41** | **-1.40** | **0.54** | **0.39** | **3.74** |
|  | ***0.008*** | ***0.009*** | ***0.405*** | ***0.024*** | ***0.262*** | ***0.411*** | ***0.002*** |
| 5 | 0.14 | -0.17 | -0.14 | 0.30 | 0.28 | 0.42 | 0.55 |
|  | *0.603* | *0.531* | *0.598* | *0.296* | *0.313* | *0.126* | *0.047* |
| 6 | -8.63 | -0.14 | 1.27 | 0.24 | -4.32 | -10.00 | 5.56 |
|  | *0.276* | *0.875* | *0.106* | *0.753* | *0.394* | *0.214* | *0.276* |
| 7 | -0.74 | -0.62 | 1.52 | -0.76 | -1.36 | -13.12 | -10.43 |
|  | *0.238* | *0.346* | *0.053* | *0.219* | *0.065* | *0.995* | *0.996* |
| **8** | **-1.81** | **0.06** | **0.41** | **4.37** | **0.45** | **-6.96** | **13.76** |
|  | ***0.511*** | ***0.981*** | ***0.654*** | ***0.269*** | ***0.784*** | ***0.958*** | ***0.917*** |
| **9** | **-2.97** | **-5.56** | **3.62** | **5.99** | **0.98** | **-1.64** | **18.89** |
|  | ***0.513*** | ***0.360*** | ***0.288*** | ***0.288*** | ***0.810*** | ***0.674*** | ***0.033*** |
| 10 | 0.22 | -0.17 | 0.14 | 1.68 | -0.14 | 1.50 | 1.30 |
|  | *0.551* | *0.634* | *0.699* | *0.001* | *0.705* | *0.001* | *0.002* |
| 11 | 0.22 | -0.82 | 0.87 | -0.59 | -0.35 | 0.00 | 0.26 |
|  | *0.476* | *0.015* | *0.006* | *0.047* | *0.250* | *0.991* | *0.399* |
| 12 | -11.10 | -0.76 | 0.12 | -0.41 | -1.10 | -12.74 | -1.94 |
|  | *0.996* | *0.240* | *0.820* | *0.458* | *0.065* | *0.996* | *0.008* |
| 13 | 1.52 | -0.13 | -2.23 | 4.49 | 0.04 | 0.41 | -0.79 |
|  | *0.029* | *0.793* | *0.002* | *0.001* | *0.933* | *0.375* | *0.104* |
| 14 | 1.63 | 0.34 | -0.83 | 0.55 | 1.91 | 2.81 | 2.25 |
|  | *0.011* | *0.445* | *0.087* | *0.229* | *0.004* | *0.001* | *0.002* |
| 15 | -1.15 | 1.17 | -0.44 | -0.07 | 0.19 | -0.31 | 0.07 |
|  | *0.001* | *0.002* | *0.170* | *0.841* | *0.547* | *0.349* | *0.832* |
| 16 | -1.58 | 0.86 | -1.77 | -1.65 | 1.59 | 2.56 | 0.23 |
|  | *0.012* | *0.076* | *0.009* | *0.005* | *0.006* | *0.001* | *0.604* |
| 17 | -0.08 | 0.49 | -0.62 | -0.32 | 0.12 | -0.48 | 0.09 |
|  | *0.773* | *0.088* | *0.032* | *0.261* | *0.667* | *0.095* | *0.748* |
| 18 | Inf | 0.00 | 0.00 | 0.00 | 0.00 | 0.00 | 0.00 |
|  | *0.000* | *0.000* | *0.000* | *0.000* | *0.000* | *0.000* | *0.000* |
| 19 | 2.41 | -0.84 | -0.08 | -1.58 | 1.58 | 1.43 | 2.67 |
|  | *0.002* | *0.085* | *0.858* | *0.013* | *0.009* | *0.010* | *0.002* |
| 20 | -0.84 | -1.64 | 0.30 | 0.61 | -1.78 | -0.99 | 1.22 |
|  | *0.039* | *0.002* | *0.418* | *0.102* | *0.001* | *0.029* | *0.006* |
| 21 | 1.06 | 0.10 | -0.29 | 3.04 | 1.38 | 3.60 | 0.25 |
|  | *0.064* | *0.842* | *0.561* | *0.003* | *0.054* | *0.001* | *0.614* |
| **22** | **-0.49** | **-1.47** | **-0.47** | **0.21** | **-0.27** | **-0.67** | **3.20** |
|  | ***0.331*** | ***0.038*** | ***0.362*** | ***0.671*** | ***0.586*** | ***0.223*** | ***0.000*** |
| 23 | 6.01 | 0.10 | 1.05 | -13.57 | -11.29 | 3.12 | 1.12 |
|  | *0.008* | *0.911* | *0.376* | *0.011* | *0.011* | *0.027* | *0.201* |
| 24 | 0.85 | -0.20 | -0.19 | -1.08 | 1.31 | 3.13 | 0.75 |
|  | *0.112* | *0.701* | *0.700* | *0.055* | *0.032* | *0.000* | *0.139* |
| 25 | -15.01 | -0.06 | -0.29 | 10.31 | 0.32 | -3.58 | -0.67 |
|  | *0.287* | *0.976* | *0.943* | *0.268* | *0.934* | *0.362* | *0.484* |
| 26 | 0.27 | 0.11 | 0.08 | 0.95 | 0.25 | 0.57 | 0.58 |
|  | *0.351* | *0.698* | *0.775* | *0.005* | *0.385* | *0.055* | *0.054* |
| **27** | **-2.97** | **-5.56** | **3.62** | **5.99** | **0.98** | **-1.64** | **18.89** |
|  | ***0.513*** | ***0.360*** | ***0.288*** | ***0.288*** | ***0.810*** | ***0.674*** | ***0.033*** |
| 28 | -4.24 | 7.41 | -0.84 | 3.82 | -0.38 | -1.52 | 0.72 |
|  | *0.038* | *0.017* | *0.260* | *0.028* | *0.533* | *0.051* | *0.328* |
| **29** | **-4.71** | **-6.97** | **3.16** | **3.92** | **4.30** | **1.66** | **19.41** |
|  | ***0.151*** | ***0.141*** | ***0.119*** | ***0.304*** | ***0.081*** | ***0.344*** | ***0.005*** |
| 30 | 0.13 | 0.40 | -0.26 | -0.03 | -0.10 | 0.01 | 0.01 |
|  | *0.607* | *0.131* | *0.309* | *0.920* | *0.701* | *0.968* | *0.957* |
| 31 | 0.72 | -1.61 | -1.75 | -9.90 | -1.91 | 3.76 | 0.33 |
|  | *0.365* | *0.118* | *0.152* | *0.022* | *0.094* | *0.042* | *0.626* |
| 32 | -0.21 | -1.45 | 0.13 | -1.59 | -0.81 | -1.66 | -0.45 |
|  | *0.568* | *0.003* | *0.720* | *0.002* | *0.046* | *0.001* | *0.231* |
| 33 | 0.79 | 0.52 | -0.65 | 1.51 | -12.27 | -0.65 | 11.21 |
|  | *0.131* | *0.256* | *0.192* | *0.024* | *0.997* | *0.206* | *0.997* |
| **34** | **-0.70** | **0.10** | **1.20** | **-0.69** | **0.38** | **-0.02** | **2.75** |
|  | ***0.135*** | ***0.827*** | ***0.042*** | ***0.127*** | ***0.395*** | ***0.967*** | ***0.000*** |
| 35 | 6.26 | -2.77 | 1.98 | 2.11 | 7.89 | -14.96 | 8.69 |
|  | *0.008* | *0.159* | *0.147* | *0.140* | *0.006* | *0.002* | *0.007* |

Table presents coefficients and *p*-values (below in italics) indicating the significance of each predictive factor,,in a logistic regression model of treatment choice, in each subject (as described in *Methods*). Coefficients shown here describe the probability that the subject would choose Drug A. Subject 18 chose the same medication for all patients during the Testing Phase. DM, diabetes mellitus. High performers are highlighted in bold.
