# Supplementary material for: Neural Correlates of Effective Learning in Experienced Medical Decision-Makers
Source: PLoS One. 2011 Nov 23;6(11):e27768. doi: 10.1371/journal.pone.0027768 (PMC3223201; doi:10.1371/journal.pone.0027768)
Supplement: Table S4 — Assessment of significant predictive factors in the training encounter set for each individual subject. Although the training scenario sequence and the outcome of each of the two available choices was held fixed across all subjects, each subject still made a unique sequence of choices and thus saw a unique set of outcomes. Theoretically, a subject could have encountered a statistical difference in efficacy with, for example, smoking status, simply through chance sampling of the fixed outcomes. Hence, we assessed whether subjects' treatment rules inadvertently reflected real trends in the Training Phase. For each subject, we performed another logistic regression of all 6 factors against Training Phase outcomes for each drug. This allowed us determine, for each subject's unique set of outcomes, whether the two treatment choices actually did have significantly different efficacy as predicted by diabetes or any of the other patient factors. This table presents p-values indicating, for each factor, the significance of the observed differences in efficacy between the two medications, based on the specific sequence of choices and outcomes seen by each individual subject during training. P-values<0.05 are indicated in boldface. This model confirmed that diabetes status predicted optimal choice (p<0.05) in 33 of 35 subjects' training sets. Subject 10 mostly chose a single drug for diabetic patients during training, so would have been unable to determine differential efficacy. Only in subjects 1 and 3 did a single treatment-irrelevant factor inadvertently achieve predictive significance during training. Subject 1 still did not develop a significant treatment rule around this factor. Subject 3 did develop a rule around the factor, as well as 3 other confirmed irrelevant factors. (DOC) [file pone.0027768.s005.doc]

**Supplementary Table S4: Assessment of significant predictive factors in the training encounter set for each individual subject.**

|  |  | **Overall** |  |  |  |  | **Factor Predictiveness** | | | | |  |  |  |
| --- | --- | --- | --- | --- | --- | --- | --- | --- | --- | --- | --- | --- | --- | --- |
| **Subject** |  | **Efficacy** |  | **Age** |  | **Sex** |  | **Sympt** |  | **Smoking** |  | **Prev MI** |  | **DM** |
|  |  |  |  |  |  |  |  | **Duration** |  | **Hx** |  | **Hx** |  | **Hx** |
|  |  |  |  |  |  |  |  |  |  |  |  |  |  |  |
| 1 |  | 0.0570 |  | **0.0439** |  | 0.0584 |  | 0.1652 |  | 0.2145 |  | 0.0895 |  | **0.0436** |
| 2 |  | 0.7074 |  | 0.4041 |  | 0.7702 |  | 0.6554 |  | 0.7608 |  | 0.5733 |  | **0.0170** |
| 3 |  | 0.7719 |  | 0.5964 |  | 0.4223 |  | **0.0463** |  | 0.3507 |  | 0.8774 |  | **0.0032** |
| 4 |  | 0.7038 |  | 0.5797 |  | 0.7514 |  | 0.9451 |  | 0.6375 |  | 0.8021 |  | **0.0025** |
| 5 |  | 0.8966 |  | 0.2189 |  | 0.6805 |  | 0.3299 |  | 0.5780 |  | 0.9621 |  | **0.0015** |
| 6 |  | 0.4858 |  | 0.5151 |  | 0.5636 |  | 0.3156 |  | 0.2548 |  | 0.6812 |  | **0.0020** |
| 7 |  | 0.7302 |  | 0.4454 |  | 0.8858 |  | 0.8590 |  | 0.9557 |  | 0.5806 |  | **0.0016** |
| 8 |  | 0.1829 |  | 0.0710 |  | 0.1393 |  | 0.8553 |  | 0.7505 |  | 0.7914 |  | **0.0284** |
| 9 |  | 0.4932 |  | 0.4866 |  | 0.6174 |  | 0.8579 |  | 0.4280 |  | 0.6592 |  | **0.0302** |
| 10 |  | 0.9999 |  | 0.8281 |  | 0.7482 |  | 0.8922 |  | 0.8934 |  | 0.6246 |  | 0.9998 |
| 11 |  | 0.8070 |  | 0.9394 |  | 0.8844 |  | 0.6032 |  | 0.6657 |  | 0.9611 |  | **0.0031** |
| 12 |  | 0.7393 |  | 0.5297 |  | 0.8588 |  | 0.8353 |  | 0.8843 |  | 0.9057 |  | **0.0019** |
| 13 |  | 0.9998 |  | 0.3193 |  | 0.9998 |  | 0.8780 |  | 0.6872 |  | 0.8760 |  | **0.0019** |
| 14 |  | 0.8196 |  | 0.3520 |  | 0.9428 |  | 0.4965 |  | 0.9882 |  | 0.7568 |  | **0.0039** |
| 15 |  | 0.6685 |  | 0.5091 |  | 0.9998 |  | 0.5522 |  | 0.6263 |  | 0.8466 |  | 0.9997 |
| 16 |  | 0.3141 |  | 0.0747 |  | 0.7229 |  | 0.4432 |  | 0.7667 |  | 0.6616 |  | **0.0032** |
| 17 |  | 0.3149 |  | 0.7347 |  | 0.8890 |  | 0.8741 |  | 0.4013 |  | 0.6414 |  | **0.0015** |
| 18 |  | 0.9740 |  | 0.4274 |  | 0.7515 |  | 0.7583 |  | 0.7254 |  | 0.9515 |  | **0.0015** |
| 19 |  | 0.9804 |  | 0.4451 |  | 0.6852 |  | 0.5305 |  | 0.2544 |  | 0.9991 |  | **0.0012** |
| 20 |  | 0.7392 |  | 0.2106 |  | 0.8681 |  | 0.9303 |  | 0.8144 |  | 0.9564 |  | **0.0246** |
| 21 |  | 0.5189 |  | 0.8234 |  | 0.7347 |  | 0.3256 |  | 0.8110 |  | 0.8931 |  | **0.0031** |
| 22 |  | 0.8746 |  | 0.3707 |  | 0.6321 |  | 0.6299 |  | 0.9177 |  | 0.8140 |  | **0.0107** |
| 23 |  | 0.8254 |  | 0.6174 |  | 0.6767 |  | 0.8723 |  | 0.9177 |  | 0.8582 |  | **0.0020** |
| 24 |  | 0.8290 |  | 0.4713 |  | 0.9363 |  | 0.8782 |  | 0.6992 |  | 0.7010 |  | **0.0016** |
| 25 |  | 0.5883 |  | 0.2993 |  | 0.8705 |  | 0.7554 |  | 0.3526 |  | 0.4534 |  | **0.0108** |
| 26 |  | 0.6714 |  | 0.3980 |  | 0.4142 |  | 0.6833 |  | 0.6244 |  | 0.9863 |  | **0.0083** |
| 27 |  | 0.7462 |  | 0.1655 |  | 0.9084 |  | 0.7395 |  | 0.9894 |  | 0.9690 |  | **0.0351** |
| 28 |  | 0.8105 |  | 0.2377 |  | 0.7860 |  | 0.3460 |  | 0.1913 |  | 0.9889 |  | **0.0024** |
| 29 |  | 0.6529 |  | 0.3712 |  | 0.8557 |  | 0.9808 |  | 0.9648 |  | 0.9020 |  | **0.0047** |
| 30 |  | 0.8342 |  | 0.8342 |  | 0.2984 |  | 0.7188 |  | 0.9641 |  | 0.9418 |  | **0.0036** |
| 31 |  | 0.4225 |  | 0.2009 |  | 0.5544 |  | 0.6429 |  | 0.6111 |  | 0.8436 |  | **0.0059** |
| 32 |  | 0.3550 |  | 0.3326 |  | 0.9547 |  | 0.3865 |  | 0.5252 |  | 0.3906 |  | **0.0024** |
| 33 |  | 0.8406 |  | 0.8382 |  | 0.3226 |  | 0.8185 |  | 0.9899 |  | 0.4898 |  | **0.0029** |
| 34 |  | 0.3357 |  | 0.1230 |  | 0.9486 |  | 0.8058 |  | 0.8301 |  | 0.5945 |  | **0.0021** |
| 35 |  | 0.9628 |  | 0.2210 |  | 0.8570 |  | 0.9901 |  | 0.9722 |  | 0.7952 |  | **0.0027** |

Although the training scenario sequence and the outcome of each of the two available choices was held fixed across all subjects, each subject still made a unique sequence of choices and thus saw a unique set of outcomes. Theoretically, a subject could have encountered a statistical difference in efficacy with, for example, smoking status, simply through chance sampling of the fixed outcomes. Hence, we assessed whether subjects' treatment rules inadvertently reflected real trends in the Training Phase. For each subject, we performed another logistic regression of all 6 factors against Training Phase outcomes for each drug. This allowed us determine, for each subject’s unique set of outcomes, whether the two treatment choices actually did have significantly different efficacy as predicted by diabetes or any of the other patient factors.

This table presents *p*-values indicating, for each factor, the significance of the observed differences in efficacy between the two medications, based on the specific sequence of choices and outcomes seen by each individual subject during training. P-values < 0.05 are indicated in boldface.

This model confirmed that diabetes status predicted optimal choice (*p* < 0.05) in 33 of 35 subjects’ training sets. Subject 10 mostly chose a single drug for diabetic patients during training, so would have been unable to determine differential efficacy. Only in subjects 1 and 3 did a single treatment-irrelevant factor inadvertently achieve predictive significance during training. Subject 1 still did not develop a significant treatment rule around this factor. Subject 3 did develop a rule around the factor, as well as 3 other confirmed irrelevant factors.
