# Supplementary material for: Neural Correlates of Effective Learning in Experienced Medical Decision-Makers
Source: PLoS One. 2011 Nov 23;6(11):e27768. doi: 10.1371/journal.pone.0027768 (PMC3223201; doi:10.1371/journal.pone.0027768)
Supplement: Table S5 — Learning Rate Estimates For High- versus Low Performers. Estimated learning rates for high-performing and low-performing subjects, using the modified Rescorla-Wagner (RW) model as described in Methods . Subjects are ordered by percentage of optimal selections during the Testing Phase. All high performers showed positive learning rates from treatment failures as well as successes. 50% of low performers showed zero learning rates from failures. Model errors reflect the difference between the treatment algorithm predicted by the RW model with the learning rates as shown, and the actual algorithm as measured by the logistic regression model of treatment choices during the Testing Phase. Errors are expressed as the angle between normalized 7-dimensional vectors corresponding to the two algorithms, in degrees. AIC's are reported for both the adapted Rescorla-Wagner Model with asymmetric learning and a traditional Rescorla-Wagner Model. Subject 18 selected the same treatment for all patients, so learning rates could not be estimated. (DOC) [file pone.0027768.s006.doc]

**Supplementary Table S5: Learning Rate Estimates For High- versus Low-Performers**

| **Subject #** | **% Optimal**  **Choices** | **Learning**  **Rate**  **(Successes)** | **Learning**  **Rate**  **(Failures)** | **Model Error**  **(degrees of divergence)** | **AIC (Asymmetric)** | **AIC**  **(Traditional)** |
| --- | --- | --- | --- | --- | --- | --- |
|  |  |  |  |  |  |  |
| *High Performers* | |  |  |  |  |  |
| 27 | 98 | 0.009 | 0.020 | 19 | 11.20 | 11.30 |
| 9 | 98 | 0.280 | 0.030 | 17 | 10.27 | 12.61 |
| 29 | 97 | 0.002 | 0.003 | 21 | 19.11 | 18.27 |
| 8 | 95 | 0.060 | 0.050 | 21 | 26.58 | 27.81 |
| 22 | 91 | 0.780 | 0.140 | 12 | 39.90 | 39.63 |
| 2 | 89 | 0.002 | 0.004 | 32 | 47.53 | 44.85 |
| 34 | 88 | 0.020 | 0.380 | 22 | 50.94 | 54.27 |
| 4 | 81 | 0.620 | 0.050 | 29 | 55.08 | 60.30 |
| 1 | 77 | 0.001 | 0 | 47 | 73.54 | 72.63 |
|  |  |  |  |  |  |  |
| *Low Performers* | |  |  |  |  |  |
| 19 | 70 | 0.780 | 0.020 | 25 | 53.34 | 75.34 |
| 14 | 69 | 0.001 | 0 | 41 | 70.26 | 82.59 |
| 33 | 67 | 0.430 | 0.820 | 44 | 84.17 | 86.76 |
| 10 | 67 | 0.001 | 0 | 47 | 80.20 | 84.34 |
| 6 | 67 | 1.000 | 0.780 | 38 | 55.93 | 64.07 |
| 35 | 64 | 1.000 | 0.970 | 25 | 50.70 | 56.70 |
| 20 | 63 | 0.850 | 0.030 | 31 | 70.30 | 83.11 |
| 26 | 61 | 1.000 | 0.480 | 57 | 90.02 | 90.27 |
| 5 | 61 | 0.990 | 0.450 | 34 | 87.71 | 87.29 |
| 24 | 55 | 0.110 | 0 | 16 | 43.56 | 88.69 |
| 11 | 55 | 0.040 | 0 | 18 | 75.19 | 89.40 |
| 23 | 53 | 0.090 | 0 | 35 | 53.31 | 92.56 |
| 31 | 52 | 0.560 | 0 | 25 | 37.32 | 90.87 |
| 21 | 52 | 0.130 | 0 | 36 | 68.48 | 92.72 |
| 16 | 52 | 0.230 | 0 | 21 | 51.81 | 92.72 |
| 30 | 50 | 0.190 | 0 | 60 | 93.90 | 92.72 |
| 18 | 50 | NaN | NaN | NaN | NaN | NaN |
| 17 | 50 | 0.300 | 0.860 | 52 | 88.29 | 89.24 |
| 3 | 50 | 0.190 | 0 | 50 | 79.21 | 92.55 |
| 28 | 48 | 1.000 | 0.350 | 66 | 86.67 | 90.37 |
| 25 | 48 | 1.000 | 0.004 | 42 | 61.32 | 91.78 |
| 15 | 48 | 1.000 | 0.070 | 55 | 86.88 | 91.06 |
| 32 | 45 | 0.980 | 0.080 | 40 | 72.53 | 88.98 |
| 13 | 44 | 0.070 | 0 | 58 | 84.00 | 91.87 |
| 7 | 41 | 0.870 | 0.070 | 55 | 65.92 | 91.57 |
| 12 | 38 | 1.000 | 0.100 | 56 | 80.86 | 79.23 |

Estimated learning rates for high-performing and low-performing subjects, using the modified Rescorla-Wagner (RW) model as described in *Methods*. Subjects are ordered by percentage of optimal selections during the Testing Phase. All high performers showed positive learning rates from treatment failures as well as successes. 50% of low performers showed zero learning rates from failures. Model errors reflect the difference between the treatment algorithm predicted by the RW model with the learning rates as shown, and the actual algorithm as measured by the logistic regression model of treatment choices during the Testing Phase. Errors are expressed as the angle between normalized 7-dimensional vectors corresponding to the two algorithms, in degrees. AIC’s are reported for both the adapted Rescorla-Wagner Model with asymmetric learning and a traditional Rescorla-Wagner Model. Subject 18 selected the same treatment for all patients, so learning rates could not be estimated.
